# Production and Purification of a Novel Xanthan Lyase from a Xanthan-Degrading *Microbacterium* sp. Strain XT11

**DOI:** 10.1155/2014/368434

**Published:** 2014-06-26

**Authors:** Fan Yang, Lan Yang, Xiaoyu Guo, Xue Wang, Lili Li, Zhicheng Liu, Wei Wang, Xianzhen Li

**Affiliations:** School of Biological Engineering, Dalian Polytechnic University, Ganjingqu, Dalian 116034, China

## Abstract

A xanthan lyase was produced and purified from the culture supernatant of an excellent xanthan-modifying strain *Microbacterium* sp. XT11. Xanthan lyase was induced by xanthan but was inhibited by its structural monomer glucose. Its production by strain XT11 is much higher than that by all other reported strains. The purified xanthan lyase has a molecular mass of 110 kDa and a specific activity of 28.2 U/mg that was much higher than that of both *Paenibacillus* and *Bacillus* lyases. It was specific on the pyruvated mannosyl residue in the intact xanthan molecule, but about 50% lyase activity remained when xanthan was partially depyruvated. Xanthan lyase was optimally active at pH 6.0–6.5 and 40°C and alkali-tolerant at a high pH value of 11.0. The metal ions including K^+^, Ca^2+^, Na^+^, Mg^2+^, Mn^2+^, and Li^+^ strongly stimulated xanthan lyase activity but ions Zn^2+^ and Cu^2+^ were its inhibitor. Xanthan lyase should be a novel enzyme different from the other xanthan lyases ever reported.

## 1. Introduction

**Figure 1:**
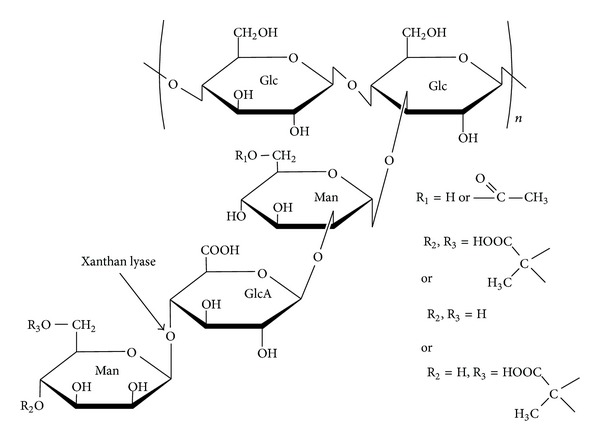
Chemical structure of xanthan. The cleavage site for xanthan lyase is indicated by solid arrows. Glc, D-glucose; Man, D-mannose; GlcA, D-glucuronic acid.

Xanthan is an important anionic heteropolysaccharide produced by* Xanthomonas campestris* pv.* campestris* and contains a cellulosic backbone with the mannosyl-glucuronyl-mannosyl side chain linked to an alternated glucosyl residue at C-3 position (Figure 1) [1, 2]. The inner mannosyl residues are acetylated and the terminal mannosyl residues are pyruvated in the side chain depending on the xanthan-producing strains and culture condition [3, 4]. Such structure makes xanthan have peculiar rheological properties, leading to a wide application in the industrial process as a thickener, gelling agent or stabilizer of emulsion and dispersion [1, 2, 5].

It has been demonstrated that the truncation of the side chain in xanthan molecule could result in some benefits from a scientific or practical point of view. The removal of the mannosyl residue in the terminal side chains could result in weaker viscosifier injected into the underground oil bearing formation [6]. The cut-off of both the terminal mannosyl and the glucuronyl residues produces a viscosifier superior to xanthan [7]. Furthermore, elicitor-active oligosaccharides produced from xanthan with the truncated side chain could block the formation of black rot lesions on cruciferous plants by inhibiting* X. campestris* pv.* campestris* [8, 9].

The variant xanthan with the truncated side chain could be produced by* X. campestris* mutants [7, 10], whereas its production level is far from what is required for practical use [11]. Therefore, because of the molecular design of xanthan, the use of relevant enzymes seems to be a preferable and promising way to obtain a tailor-made xanthan with specific and desired properties [12]. Differing from other polysaccharide lyases acting on the polysaccharide backbone, xanthan lyase could cleave the linkage between the terminal mannosyl and the glucuronyl residues on the side chain by a *β*-elimination reaction, introducing a double bond between C4 and C5 of the uronosyl residue and subsequently might be exploited for further chemical modification [13]. However, xanthan lyase was purified from both* Paenibacillus alginolyticus* XL-1 and* Bacillus* sp. GL1 so far [14, 15], in which the production of xanthan lyase was too low to meet the demand for the structural modification of xanthan.

In our previous work, an excellent xanthan-degrading bacterium* Microbacterium* sp. XT11 was isolated [9], which produced high activity of extracellular xanthan-degrading enzymes and could efficiently fragment xanthan to form the bioactive oligosaccharide [8, 9]. The endoxanthanase cleaving the backbone linkage of the xanthan has been purified [8], whereas the xanthan lyase was not characterized and the way to modify the terminal side chains of xanthan remained unclear. In this paper, the xanthan lyase produced by* Microbacterium* sp. XT11 was purified and characterized. It is valuable for modifying xanthan molecules to extend its application in oil enhancing recovery and bioactive oligosaccharide production.

## 2. Materials and Methods

### 2.1. Microorganism

*Microbacterium* sp. XT11 was isolated from soil for xanthan degradation in our lab and cultured in the xanthan medium at 30°C as described elsewhere [9]. The xanthan medium consists of (per liter) 3 g xanthan and 3 g yeast extract in the mineral salt solution, pH 7.0. The mineral salt solution contains (per liter) 50 mg K_2_HPO_4_, 800 mg NaCl, 25 mg MgSO_4_ *·*7H_2_O, and 700 mg KNO_3_.

### 2.2. Optimum Culture Condition and Xanthan Lyase Production

The influence of carbon source on xanthan lyase production was performed by incubating the strain XT11 in the mineral salt solution (pH 7.0) supplemented with 0.3% yeast extract and 0.3% different carbohydrates including xanthan, glucose, sucrose, fructose, starch, and xanthan plus 0.2% glucose at 30°C and 150 rpm. The relationship between the nitrogen source and xanthan lyase production was determined by culturing the strain XT11 in the mineral salt solution (pH 7.0) with 0.3% xanthan and varied nitrogen sources including 0.3% yeast extract, peptone, ammonium sulfate, tryptone, and corn extract, respectively. The optimal pH for the production of xanthan lyase was evaluated in the xanthan medium at pH range of 2.0–10.0.

To determine the time-course of the strain XT11 cultured in the xanthan medium, samples were collected every 6 h to measure cell growth and xanthan lyase activity, in which the xanthan lyase was determined in the supernatant after centrifugation at 10,000 g and 4°C for 30 min. All the experiments were performed thrice.

In total, 5 L of the xanthan medium distributed in ten 2 L flasks was inoculated with the overnight culture of* Microbacterium* sp. XT11 at a ratio of 1 : 50. After incubation for 18 h the supernatant was obtained by centrifuging the culture at 10,000 g and 4°C for 30 min and used for enzyme purification.

### 2.3. Purification of Xanthan Lyase

A representative process for the purification of xanthan lyase was carried out as follows. All operations were performed at 4°C unless otherwise specified.

(i) Ammonium sulfate fractionation: the culture supernatant was adjusted to pH 6.0 and centrifuged at 10,000 g for 30 min. Ammonium sulfate was added portion-wise to a final concentration of 30–60% saturation, and the mixture was gently stirred overnight. The suspension was centrifuged at 10,000 g for 30 min. The recovered precipitate was dissolved in 20 mM potassium phosphate buffer (KPB) at pH 6.0 and dialyzed against the same buffer with three changes of the buffer, which was used as the crude enzyme solution.

(ii) Hydrophobic interaction chromatography: to the crude enzyme solution, solid (NH_4_)_2_SO_4_ was added to a final concentration of 0.8 M. After being filtered through a 0.22 *μ*m durapore membrane (Millipore Corporation, USA), the enzyme solution was passed through a hydrophobic interaction chromatography (HIC) column packed with 5 mL phenyl Sepharose resin (GE Healthcare, Sweden) previously equilibrated with 20 mM KPB at pH 6.0. Protein was eluted with a linear gradient of 0.8 to 0 M (NH_4_)_2_SO_4_ in 20 mM KPB (pH 6.0) at a flow rate of 2.0 mL/min in a total volume of 50 mL. The fractions were collected and protein was detected by UV light absorption at 280 nm. The active xanthan lyase fractions were pooled and dialyzed overnight at 4°C against 20 mM KPB (pH 6.0).

(iii) Anion-exchange chromatography: the HIC pool was applied to an anion-exchange chromatography (AEC) column (5 mL Q Sepharose FF resin, GE Healthcare, Sweden). Protein was eluted with a linear gradient of 0 to 1.0 M NaCl in 20 mM KPB (pH 6.0) at a flow rate of 1 mL/min in a total volume of 50 mL. Xanthan lyase-containing fractions were pooled and concentrated using a stirred ultrafiltration cell (Millipore Corporation, USA) over a 10-KDa molecular weight cut-off membrane.

For every purification step, fractions containing the target protein were determined by SDS-PAGE analysis.

### 2.4. Characterization of Xanthan Lyase

To assess the temperature optimum for xanthan lyase activity, *V*_max⁡_ was determined by Lineweaver-Burk plot in 100 mM phosphate buffer (pH 6.0) at different temperatures ranging from 20°C to 70°C [16]. The thermostability of xanthan lyase was determined by assaying the *V*_max⁡_ after the xanthan lyase was incubated in 100 mM phosphate buffer (pH 6.0) at various temperatures (20–60°C) for 30 min. The optimal pH for xanthan lyase activity was assayed by calculating the *V*_max⁡_ from Lineweaver-Burk plot in 100 mM phosphate buffer at different pH [16]. To measure the pH stability, *V*_max⁡_ assays were performed using the enzyme sample that was preincubated at different pH for 2 h.

Under the optional temperature and pH condition, the enzyme activity was measured with the addition of 10 mM different metal ions, including K^+^, Ca^+^, Na^+^, Mg^2+^, Zn^2+^, Cu^2+^, Mn^2+^, and Li^+^ in the reaction system, respectively. All experiments were done in triplicate.

### 2.5. SDS-PAGE Analysis

The sodium dodecyl sulfate-polyacrylamide gel electrophoresis (SDS-PAGE) was carried out as follows. Aliquots of the protein samples purified at different stages were loaded onto the stacking gel contained 5% polyacrylamide and separated in the resolving gel contained 12% polyacrylamide. Protein was visualized by coomassie brilliant blue R-250 or silver staining.

### 2.6. Purification and Chemical Modification of Substrate Xanthan

Xanthan was purified as described previously [17]. Briefly, the food-grade xanthan was dissolved in the distilled water and diluted to 1 g/L. Xanthan was precipitated by adding the equal volume of ice-cold absolute ethanol in the presence of NaCl (100 g/L) after stirring for 2 h at 4°C. The precipitate was collected by filtration and washed with ethanol-water mixture up to 100% in ethanol. The purified xanthan was lyophilized for the following test.

The chemical modification of xanthan was performed using the following procedures. The solution containing 5 g/L of the purified xanthan in 5 mM trifluoroacetic acid was heated at 100°C for 1.5 h to remove the pyruvic acetyl groups [18]. The purified xanthan (2.5 g/L) in 0.1 M NH_4_OH was incubated at 60°C for 1 h to remove the acetyl groups [19]. Both the depyruvated and the deacetylated xanthan were dialyzed against the distilled water and lyophilized, respectively.

### 2.7. Enzyme Assay

All assays were done in triplicates, and the mean values were presented.

The activity of xanthan lyase was assayed in the reaction mixture containing 0.5 mg/mL of the purified xanthan in 100 mM potassium phosphate buffer (pH 6.0) and appropriately diluted xanthan lyase. After incubation at 40°C for 10 min, the increase in the absorbance at 235 nm was determined. Appropriate controls for the absorbance decrease with addition of the heated xanthan lyase (100°C, 5 min) were included. One unit of xanthan lyase activity was defined as the amount of enzyme that produced an increase of 1.0 in the absorbance at 235 nm per min under the above condition.

### 2.8. Chemical Analysis

The acetyl content of xanthan was measured according to the method of Hestrin [20] with acetylcholine as a reference. To determine the pyruvate content of xanthan, 0.01 g xanthan was hydrolyzed in 5 mL HCl (1 M) at 100°C for 3 h, and the free pyruvate was determined as described by Sloneker and Orentas [21] with sodium pyruvate as the reference. Protein concentration was measured using the Bio-Rad protein assay kit (Bio-Rad, USA). Cell growth was determined by optical density (O.D.) at 600 nm.

## 3. Results

### 3.1. Production of Xanthan Lyase by* Microbacterium* sp. XT11

**Figure 2:**
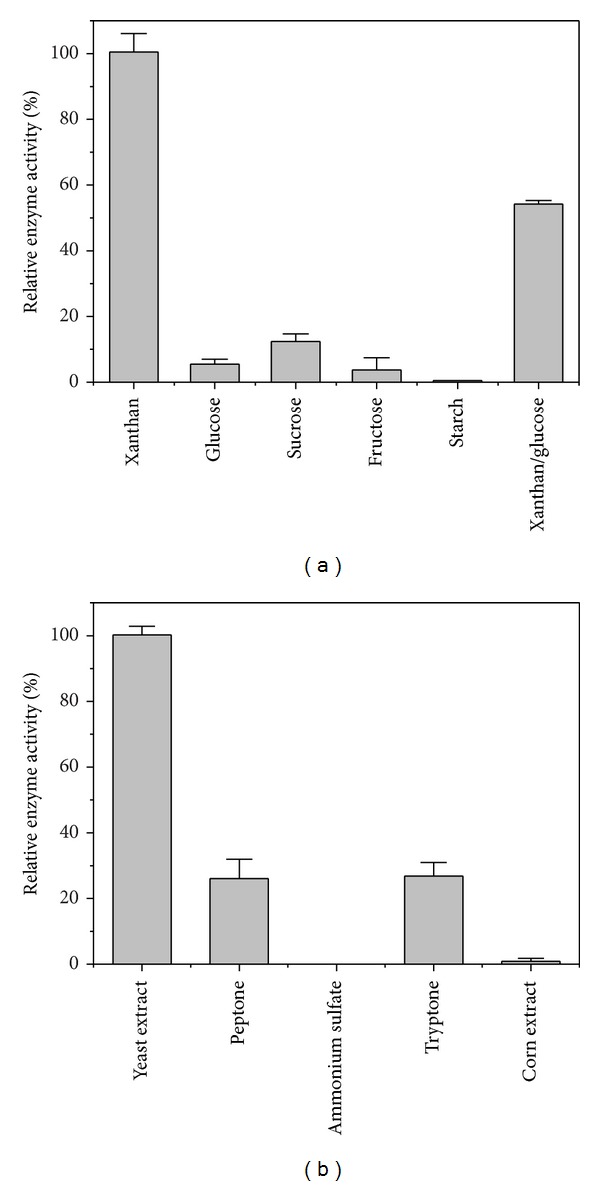
Xanthan lyase production when* Microbacterium* sp. XT11 was cultured in the medium with different carbon sources (a) and various nitrogen sources (b) shaking at 30°C.

The effect of carbon sources and nitrogen sources on the production of xanthan lyase was determined as shown in Figure 2. The highest xanthan lyase activity was obtained when xanthan was used as carbon source, while much lower titers of xanthan lyase were produced with all other tested carbohydrates (Figure 2(a)). When 0.2% glucose was supplemented in the xanthan medium, less than 60% activity remained. The maximal activity of xanthan lyase was detected in the xanthan medium with yeast extract as nitrogen source compared with the other tested nitrogen sources (Figure 2(b)). Almost no xanthan lyase activity could be detected when ammonium sulfate or corn extract was used as nitrogen source, and only about 25% enzyme activity was obtained in the xanthan medium with peptone or tryptone as nitrogen source.

**Figure 3:**
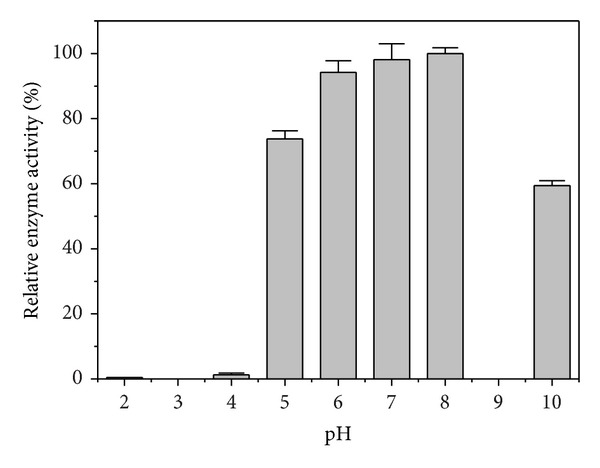
Influence of initial pH on xanthan lyase production by* Microbacterium* sp. XT11 cultured in the xanthan medium at 30°C and 150 rpm.

The influence of the initial pH in the xanthan medium on xanthan lyase production was shown in Figure 3. The maximal enzyme activity was detected when strain XT11 was cultured in the xanthan medium at pH range of 6.0–8.0. No xanthan lyase activity could be detected in the culture fluid of* Microbacterium* sp. XT11 when the initial pH of the xanthan medium was lower than pH 4.0, but more than 60% enzyme activity was obtained even though the strain XT11 was incubated at pH 10.0.

**Figure 4:**
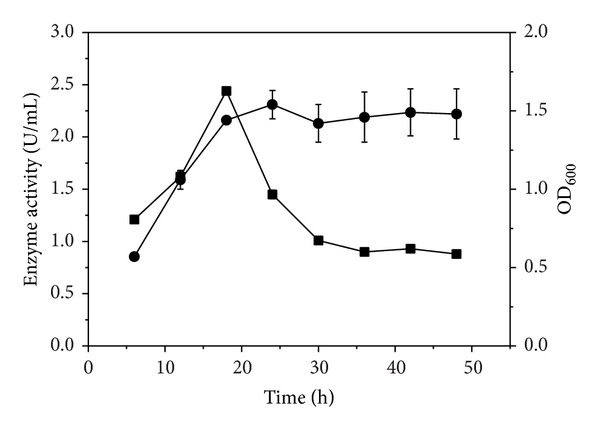
Time-course of cell growth (●) and xanthan lyase activity (■) when* Microbacterium *sp. XT11 was cultured in 0.3% xanthan medium at 30°C and shaking at 150 rpm.

The time-course of cell growth and xanthan lyase production was shown in Figure 4. In the case that 0.3% xanthan was used as carbon source, the maximum enzyme activity (2.43 ± 0.05 U/mL) was obtained after the strain XT11 was cultured for 18 h, whereas the enzyme activity was decreased significantly when the culture time was extended from 18 h to 48 h. The OD_600 nm_ tended to be stable and not to be increased obviously after the strain XT11 was cultured for 18 h. Therefore, the culture supernatant harvested at 18 h was ready for the subsequent purification of xanthan lyase.

### 3.2. Purification of Xanthan Lyase

**Figure 5:**
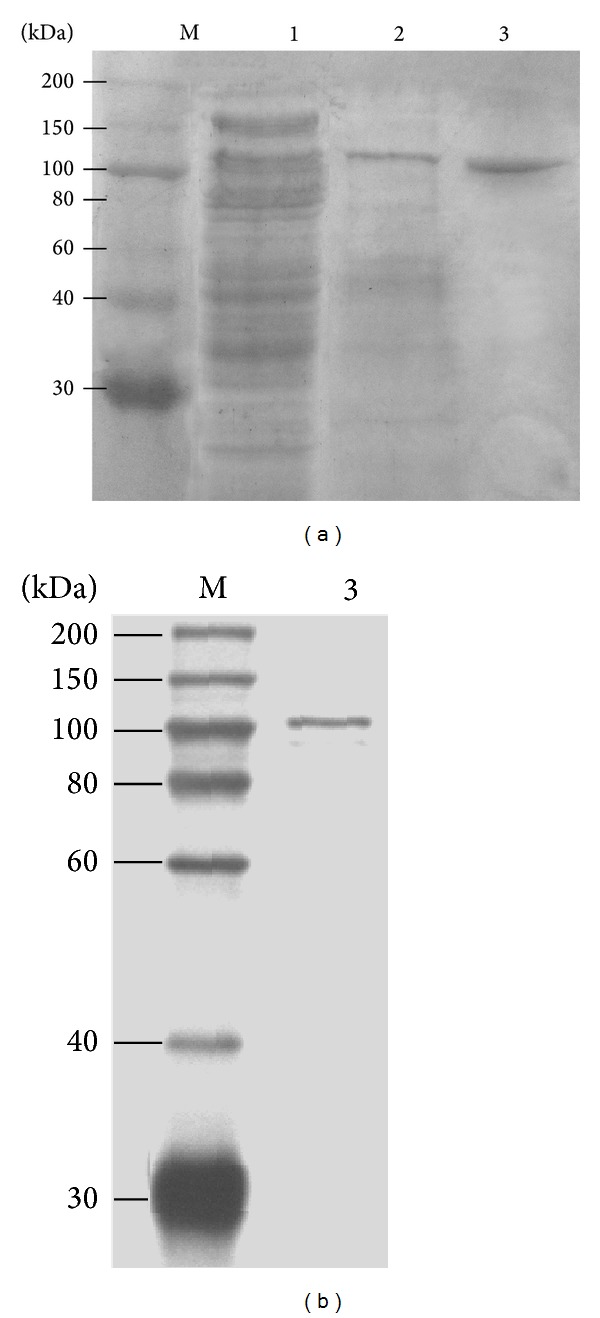
SDS-PAGE analysis of the samples from various steps of xanthan lyase purification by staining with coomassie brilliant blue (a) and the purified xanthan lyase by silver staining (b). Lane 1: ammonium sulfate precipitation sample; lane 2: the eluted protein solution after the phenyl Sepharose column; lane 3: the final xanthan lyase product after the Q Sepharose FF column; and lane M: the protein marker.

Purification was performed from the cell-free culture broth of* Microbacterium* sp. XT11 with a specific activity of 2.2 U/mg. The results were summarized in Table 1. In the first step, ammonium sulfate precipitation gave a sample of 2.7 U/mg with a protein recovery of 75.6%. When the crude enzyme solution was applied on the HIC column, the protein containing xanthan lyase was eluted from the phenyl Sepharose column at approximately 0.2 M (NH_4_)_2_SO_4_ in one fraction. After being dialyzed against 20 mM KPB, the HIC pool was applied to Q Sepharose FF column and the enzyme fraction was eluted at 0.3 M NaCl. The preparation gave a total protein recovery of 50.4% in a purification fold of 12.8, and the final product had a specific activity of 28.2 U/mg. As shown in Figure 5(a), samples from each purification step were analyzed by SDS-PAGE stained with coomassie brilliant blue and the impurities were gradually removed in the purification processes. To confirm the purity of xanthan lyase, the silver staining of the gel containing the purified enzyme was performed as shown in Figure 5(b).

### 3.3. Enzymatic Properties of Xanthan Lyase

The molecular mass of the xanthan lyase purified from* Microbacterium* sp. XT11 was 110 kDa as determined by both SDS-PAGE (Figure 5) and gel permeation chromatography (Sephacryl S-200HR) (data not shown).

**Figure 6:**
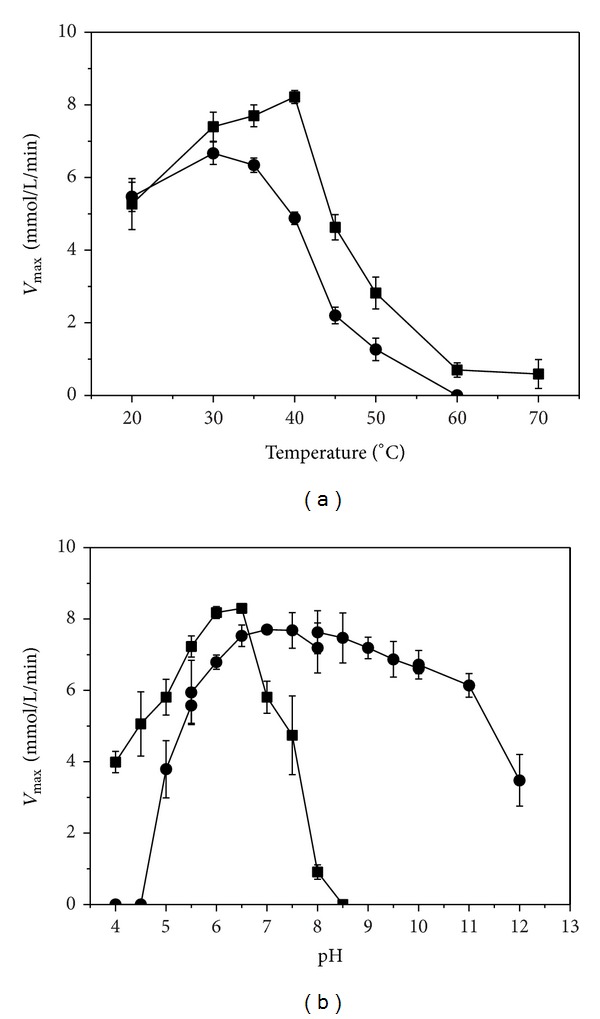
*V*_max⁡_ determined from Lineweaver-Burk plot as a function of temperature (a) and pH (b) for the reaction of* Microbacterium* sp. XT11 xanthan lyase with xanthan as substrate. The temperature/pH optimum was symbolized as “■” and the thermostability/pH stability was marked as “●”.

The xanthan lyase was active at temperature ranging from 20°C to 40°C, while almost no activity could be detected at 50°C. The maximum *V*_max⁡_ was observed at 40°C (Figure 6(a)). Xanthan lyase was relatively stable at temperature below 40°C, as high *V*_max⁡_ was observed when the enzyme was incubated at selected temperatures for 30 min (Figure 6(a)). The xanthan lyase showed the highest *V*_max⁡_ at pH range of 6.0–6.5 but reduced substantially beyond this pH range (Figure 6(b)). The enzyme was stable when the samples were held at pH range of 5.5–11.0 for 2 h (Figure 6(b)). The xanthan lyase activity was detected in the presence of different metal ions. As shown in Table 2, the xanthan lyase activity was significantly enhanced by the metal ions including K^+^, Ca^2+^, Na^+^, Mg^2+^, Mn^2+^, and Li^+^ but was intensely inhibited by Zn^2+^ and almost completely inhibited by metal ion Cu^2+^. The xanthan lyase exhibited a typical Michaelis-Menten kinetics, and the values of *K*_*m*_ and *V*_max⁡_ for xanthan were 4.23 mg/mL and 8.22 *μ*mol/L/min, respectively, under the optimal pH and temperature conditions.

To examine the substrate specificity, xanthan lyase was incubated at 40°C for 10 min in the mixture containing 100 mM potassium phosphate buffer (pH 6.0) and various substrates. The lyase activity could be detected in the reaction system with xanthan as enzymatic substrate, but there was no activity found when the other polysaccharides including alginate, heparin, hyaluronan, and pectin were used as substrate. To determine the effect of the pyruvyl and acetyl group on xanthan lyase activity, the crude xanthan substrate was purified and the acetylation and pyruvation extent of xanthan was determined as 9.7% and 15.3% respectively. All the acetyl groups could be removed when xanthan was deacetylated, and only a half of the pyruvic groups in xanthan were removed by depyruvation treatment (Table 3). As shown in Table 3, the xanthan lyase activity toward native xanthan and acetate-free xanthan was much higher than that toward the pyruvate-free xanthan and pyruvate/acetate-free xanthan.

## 4. Discussion

The truncation of the side chain had a dramatic effect on the viscometric properties of xanthan [22]. However, those tailor-made xanthans with different truncated side chains are produced at low yield by genetic engineering mutants [11]. Therefore, the enzymatic modification on the side chain is expected to be a preferable method to obtain such truncated xanthan with different physicochemical and physiological functions for specific purpose [12]. So far, an important xanthan-modifying enzyme, xanthan lyase, has been obtained from xanthan-degrading strains* P. alginolyticus* XL-1 and* Bacillus* sp. GL1 [14, 15]. However, the results showed that their xanthan lyase production was greatly decreased when the xanthan-degrading strains were purified from the mixed cultures they originated from [23, 24]. To overcome such a reduction in lyase production, a novel xanthan-degrading strain* Microbacterium* sp. XT11 was isolated in our lab [9]. One of the xanthanase (endo-1,4-*β*-D-glucanase) catalyzing the hydrolysis of the main chain of xanthan has been purified with a high specific activity [8]. All results suggested that the strain XT11 was a potential powerful tool for xanthan modification [8, 9, 25]. However, as an important xanthan-modifying enzyme, xanthan lyase has not been characterized yet, resulting in the application in truncation xanthan to be still in its infancy.

The production and characterization of xanthan lyase were studied in this paper. The data in Figure 2(a) showed a xanthan induction of xanthan lyase in* Microbacterium* sp. XT11. Furthermore, a decreased production of xanthan lyase was observed when glucose was used together with xanthan as carbon source, suggesting that the structural monomer of xanthan showed a repressive activity against its induction of xanthan lyase. It is unbelievable that the xanthan lyase production was induced by xanthan because it is too large to enter a bacterial cell. However, the similar result was obtained in the production of xanthan lyase by* P. alginolyticus* XL-1, in which the enzymatic degradation intermediate was presumed as the true xanthan lyase inducer [15]. Such an intermediate presumably is from the side chain of xanthan because *β*-1,4-glucan backbone remained intact in the culture of* P. alginolyticus* XL-1 [15].

The xanthan lyase was purified from the culture supernatant by a three-step procedure including ammonium sulfate precipitation, hydrophobic chromatography, and Q anion-exchange chromatography. The purified xanthan lyase is significantly different from the reported xanthan lyases in molecular size and belongs to the large polysaccharide lyase family, the molecular mass (110 kDa) of which is much larger than the reported enzymes with molecular mass at the range of 75 to 85 kDa in this group [14, 15]. A large xanthan lyase with 97-kDa was also obtained from* Escherichia coli* transformed with* xalB* gene of* Bacillus* sp. GL1, while it was converted to a low-molecular-mass enzyme (75-kDa) after storage at 4°C [26]. It was suggested that the xanthan lyase in* Bacillus* sp. GL1 is first secreted as a precursor (97 kDa) and then processed into a mature form (75 kDa) through excision of a C-terminal protein fragment with a molecular mass of 22 kDa [26], whereas such maturation could not be found in the culture of* Microbacterium* sp. XT11. The gene cloning of xanthan lyase is being processed to understand such a difference and its favored effect in xanthan modification. However, it is not easy to amplify this gene from* Microbacterium* sp. XT11 with a high GC content (69%) and an unclear genetic background, although about 40 entries for xanthan lyase proteins were found in NCBI database.

There was no significant difference in the optimal pH for xanthan lyase activity between the lyase from strain XT11 and other reported enzymes [14, 15]. The xanthan lyase was stable over a wide pH range, and about 90% of its total activity remained even after the lyase was held at pH 11.0 for 2 h, which is significantly different from other reported lyases [14] and similar to alkaliphilic alginate lyase from* Saccharophagus* sp. Myt-1 [27]. Different from* Bacillus* lyase being affected slightly by metal ions [14], the activity of xanthan lyase from strain XT11 was strongly enhanced by all tested metal ions except Zn^2+^ and Cu^2+^. The ion Cu^2+^ showed almost complete inhibition on xanthan lyase from strain XT11, while causing a 70%* Paenibacillus* enzyme activity drop [15]. In particular, the production of xanthan lyase by strain XT11 (2.2 U/mg) was much higher than that by other two reported xanthan-degrading strains (0.134 U/mg for* Bacillus *sp. GL1 and 0.10 U/mg for* P. alginolyticus* XL-1) [14, 15], indicating that the pure* Microbacterium* strain was competent for xanthan modification. Furthermore, the xanthan lyase from* Microbacterium* sp. XT11 had a higher specific activity (28.2 U/mg) in contrast with the reported data (6.28 U/mg for the* Bacillus* enzyme and 2.62 U/mg for the* Paenibacillus* enzyme) [14, 15].

Like the xanthan lyases produced by* Paenibacillus* and* Bacillus* [14, 15],* Microbacterium* xanthan lyase was active on the intact xanthan and was not associated with endoglucanase [8]. Thus, its true substrate probably is the intact xanthan rather than the xanthan-derived oligosaccharides. The xanthan lyase activity toward the depyruvated xanthan was significantly lower than that toward the native xanthan (Table 3), suggesting that the* Microbacterium* xanthan lyase was specific for pyruvated mannosyl residues. This finding is similar to the enzymes described by Ruijssenaars et al. [12] and Hashimoto et al. [26]. However, about half of the activity was detected when the depyruvated xanthan was used as substrate to measure the xanthan lyase from* Microbacterium* sp. XT11. This result is comparable with the fact that only a half of pyruvyl residual in xanthan molecule was removed in the acidic treatment (Table 3), which was mild to prevent the cleavage of glycosidic bonds. It is different from the* Paenibacillus* lyase that is not active on the partially depyruvated xanthan [15]. It is clear that the* Microbacterium* enzyme prefers pyruvated xanthan to nonpyruvated xanthan. As shown in Table 3, the acetyl group in xanthan molecules has no effect on xanthan lyase activity.

## 5. Conclusions

A xanthan lyase was produced and purified from the xanthan-degrading* Microbacterium* sp. XT11, which is the large polysaccharide lyase. The* Microbacterium* xanthan lyase was produced maximally when xanthan was used as carbon source, while its production was inhibited by glucose. The xanthan lyase has a higher specific activity and was stable at a wider pH range of 5.5–11.0. Most of the tested metal ions strongly stimulated xanthan lyase activity but ions Zn^2+^ and Cu^2+^ were its inhibitor. Xanthan lyase from* Microbacterium* sp. XT11 was specific on the pyruvated mannosyl residue in the intact xanthan molecules but about 50% lyase activity remained when xanthan was partially depyruvated. All data suggested that the xanthan lyase was a novel enzyme differing from the other xanthan lyases ever reported.

## Figures and Tables

**Table 1:** Purification of xanthan lyase from the xanthan-grown culture of *Microbacterium* sp. XT11.

Purification steps	Lyase activity (U)	Total protein content (mg)	Specific activity (U/mg)	Yield %	Purification fold
Cell-free culture broth	253.3	115	2.2	100	1
(NH_4_)_2_SO_4_ precipitation	191.4	71.3	2.7	75.6	1.2
Phenyl Sepharose	150.3	28.4	5.3	59.3	2.4
Q Sepharose FF	126.7	4.5	28.2	50.4	12.8

**Table 2:** Influence of metal ions on xanthan lyase activity. The assays were performed at 40°C and pH 6.5 and the residual enzyme activity was expressed as the percentage against a control without metal ion addition.

Ions	Control	K^+^	Ca^2+^	Na^+^	Mg^2+^	Zn^2+^	Cu^2+^	Mn^2+^	Li^+^
Relative activity (%)	100	196.3	194.4	192.1	186.6	53.5	19.0	152.6	191.7

**Table 3:** Influence of the various xanthan on activities of xanthan lyase produced by* Microbacterium* sp. XT11.

Xanthan samples	Acetylation (%)	Pyruvation (%)	Relative activity (%)
Native	9.7	15.3	100
Acetate-free	0	16.2	94.5
Pyruvate-free	10.2	8.0	48.8
Pyruvate-/acetate-free	0	8.7	47.9
